# Concomitant Borreliosis and Invasive Amoebiasis: A Rare Case with Suspected Sexual Acquisition

**DOI:** 10.3390/idr18040084

**Published:** 2026-08-07

**Authors:** Luisa Cavalletto, Sara Valpione, Chiara Giraudo, Claudia Mescoli, Valeria M. Besutti, Liliana Chemello

**Affiliations:** 1 Clinica Medica 5, Internal & Hepatologic Unit, University-Hospital of Padua, 35128 Padova, Italy; luisa.cavalletto@unipd.it; 2 Molecular Oncology Group, Cancer Research UK Manchester Institute, University of Manchester, Manchester SK10 4TG, UK; sara.valpione@cruk.manchester.ac.uk; 3 Radiology OSA Unit, Department of Cardiac Thoracic and Vascular Sciences and Public Health, University of Padua, 35100 Padova, Italy; chiara.giraudo@unipd.it; 4 Pathology Unit, Department of Medicine-DIMED, University-Hospital of Padua, 35128 Padova, Italy; claudia.mescoli@aopd.veneto.it; 5 Microbiology Unit, Parasitology Section, Department of Medicine-DIMED, University-Hospital of Padua, 35128 Padova, Italy; valeria.besutti@aopd.veneto.it; 6 Department of Medicine-DIMED, University of Padua, 35100 Padova, Italy

**Keywords:** pediculosis, amoebiasis, zoonosis, *Entamoeba histolytica*, allergic edema, sexual acquisition disease, borreliosis, relapsing fever, liver abscess

## Abstract

Background: The movement of endemic diseases from tropical and disadvantaged areas is gradually spreading to developed countries as well. Amoebiasis, in particular, represents a significant threat to public health, amplified by globalization and increasing contact between humans and animals or vectors. In this way, atypical diseases may emerge in our country, also through an unusual mode of transmission—likely sexual, as “sexually transmitted enteric (STE) diseases”. Objective: We report and discuss the clinic approach to a rare case of dual human infestations by ecto- and endo-parasites with multiple liver abscesses presentation in a young truck driver residing in Northern Italy. Methods: The patient underwent a comprehensive screening with laboratory and microbiological tests, and CT images. Results: In July, the patient was admitted to our hepatology unit following the ultrasound identification of two hypoechoic lesions in the right lobe of the liver. In his medical history, in April, after a seemingly harmless infestation of body lice, effectively treated, he suffered from a prolonged and debilitating episode of acute diarrhea, lasting a month. This status was also accompanied by intestinal cramps, weight loss, and recurring fever episodes. Afterwards also appeared a Quincke’s-like angioedema involving the lips and mouth mucosa, with evening time exacerbation. Conclusions: The differential diagnosis of this complex and unusual clinical picture, together with the temporal evolution of the patient’s signs and symptoms, led us to hypothesize the presence of concomitant infections. These were most likely borreliosis and a STE disease, the latter ultimately diagnosed as amebiasis with hepatic invasion, allowing appropriate treatment and a favorable clinical outcome.

## 1. Introduction

Amoebiasis, caused by *Entamoeba histolytica*, is the second leading cause of parasitic death, especially in developing countries such as some countries in the middle east and eastern Asia, central and meridional America and occidental-meridional Africa, where water and food are easily contaminated with feces containing *E. histolytica* cysts, due to poor sanitation, [1,2]. However, a growing challenge in many developed countries is the increasing incidence of invasive amoebiasis, particularly as a STE infection, which may occur through direct sexual contact among individuals practicing oro-anal sex, either in heterosexual or homosexual relationships [3]. In this context, asymptomatic infected individuals from at-risk regions constitute the main reservoir of *E. histolytica*, acting as contagious carriers and also spreading the infection to unaffected areas around the world, where the diagnosis is frequently overlooked [4].

Furthermore, the often degraded environment in which sex workers live is never free from the risk of transmission of other pathogens. Among these, some sexually transmitted infections (e.g., chlamiosis, syphilis, and HIV) certainly play a preponderant role. However, we must also consider some parasitic infestations that may act as vectors and selectively transmitted insidious bacterial infections, which are now spreading everywhere, including in Italy [5]. Body lice, although considered harmless, can cause serious cases of borreliosis, especially in frail individuals already suffering from other comorbidities. Relapsing fever (RF), caused by *Borrelia recurrentis* and *B. miyamotoi,* is transmitted, respectively, by the bite of parasites of the species *Pediculus humanus corporis*, which live and lay eggs in clothing, moving to the skin to feed [6], and *Ixodes ricinus*, a tick present in mountainous and wooded areas of northern Italy [7]. These infected vectors are reaching every continent, even carrying new species, which are more difficult to diagnose due to the specific cross-reactivity of antigens [8], and even cause co-infections of parasites [9,10] among the *Borrelia* species (spp.).

We have described a complex clinical case, characterized by a diagnostic scenario that was challenging within our regional context due to the concurrent presence of a zoonosis caused by human ecto- and endo-parasites. This infestation—potentially sexually acquired—gave rise to an episode suggestive of borreliosis, followed by invasive amoebiasis with liver abscess formation. The chronological reconstruction of events and the search for risk factors guided us through a diagnostic process, supported by a precise anamnesis, specific diagnostic tests, and effective treatment.

## 2. Case Presentation

A 41-year-old man, recently diagnosed with Quincke-like angioedema, presented to our hospital in July to investigate the etiology of newly developed hepatic abscesses. The patient reported, about 2 months before, the onset of intermittent edema episodes of the lips and oral mucosa, sometimes accompanied by fever (within 38 °C). This swelling was associated with arthralgia of the small joints of the hands and feet, and diffuse myalgia, which had already led to a rheumatological consultation, after a failed 3-day trial of antibiotic therapy (ABT) prescribed by his GP. These symptoms tended to get worse in the evening and during the night, and were not associated with itching, but with chills, fever and headache. The patient received a second course of empirical ABT (azithromycin, 500 mg tablets, once daily) for 5 days, associated with steroids (methylprednisolone 16 mg tablets, one daily dose for 3 days, de-escalate to 8 and 4 mg for a total of another 6 days), with the disappearance of symptoms occurring by the end of May.

After hospital admission in our hepatologic unit, the patient underwent a complete laboratory profile and some specific microbiological diagnostic tests, as reported in Table 1. Moderate liver enzyme alterations with leukocytosis, mild anemia, and a modest increase in the C-reactive protein (CRP) appeared. The normal eosinophil count and level of C1 inhibitor were inconsistent with Quincke’s edema or a suspected helminth infestation, prompting us to investigate alternative causes for the patient’s clinical presentation.

**Figure 1:**
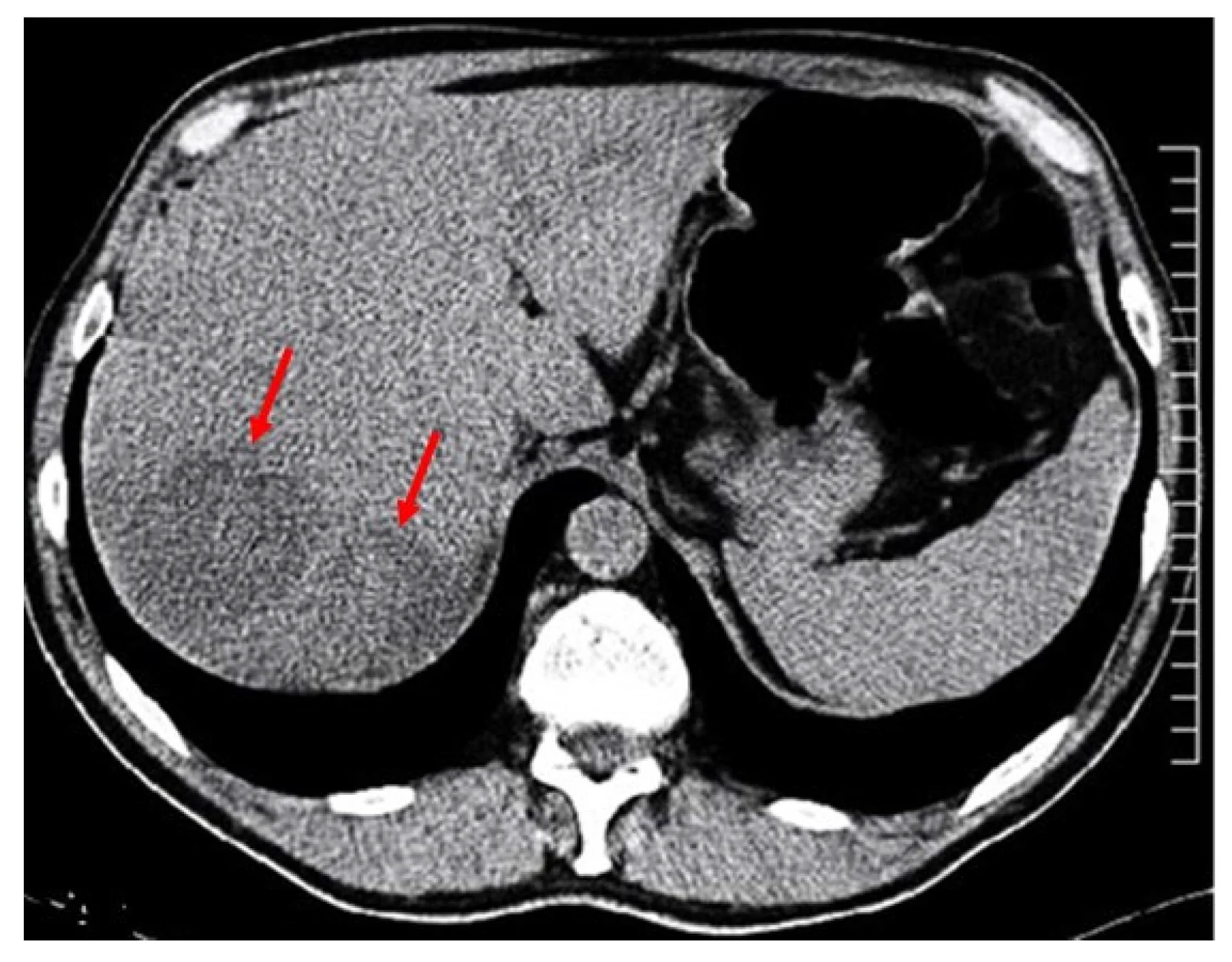
Two large hypodense liver lesions, each 8 and 6 cm in diameter, are visible in the seventh segment of the right lobe, highlighted by red arrows.

The two hypoechoic areas diagnosed by abdominal-US in the VII hepatic segment, measuring 8 and 6 cm in diameter, respectively, were confirmed with an abdominal CT scan (Figure 1).

The roundish liver lesions were soft capsulated and hypointense, and had irregular contours, being rather inhomogeneous, like necrotic liquefactive lesions. The largest one was aspirated extemporaneously by needle prick and contained a slightly thick brown necrotic fluid that, upon examination under a light microscope, revealed inflammatory blood cells with pyocytes. Bacterial and fungal cultures were negative, and no cysts were isolated. No Plasmodium species were detected, and tuberculosis was also ruled out with rapid molecular tests (NAAT).

Upon further review of the medical history, the patient reported a body louse infestation in April, which was successfully treated with a pyrethrin- and piperonyl butoxide-containing shampoo, together with decontamination of clothing and bed linens by machine washing at 65 °C. Shortly thereafter, he developed an episode of acute diarrhea associated with severe abdominal pain, which resolved spontaneously within 2–3 weeks.

Upon further questioning, the patient also recalled that the first episode of oral edema and polyarthralgia had occurred during the same period and was accompanied by recurrent febrile episodes preceded by chills and worsening headache. Moreover, he reported that the most severe episode of edema developed immediately after the first ABT course.

The patient came from a province in Northern Italy and spent part of the year traveling throughout Europe for work as a truck driver. He did not smoke and consumed alcohol only occasionally; nor did he use drugs or other narcotic substances. He enjoyed the outdoors and lived in a wooded area, but did not recall having been bitten by a tick recently. He had never traveled to tropical countries, nor had he had direct contact with animals in the woods. His family history did not confirm cases of chronic liver disease, such as cirrhosis, or hepatocellular carcinoma (HCC) among close relatives. Physical examination revealed marked weight loss (from 75 to 63 kg) over the preceding four months, associated with mild, fluctuating febrile episodes, as well as profound asthenia, hyporexia and headache. No overt jaundice was observed.

Considering the possibility of multiple causes for the various patient symptoms, and paying particular attention to reported episodes of recurrent fever, we investigated the presence of louse- or tick-borne infectious diseases, finding significant antibody positivity for *Borrelia* spp. Microbiological investigations resulted in IgG and IgM positivity by indirect chemiluminescence immunoassay (CLIA) specific for *B. burgdorferi* (LIAISON Borrelia Quant IgM and IgG tests; DIASORIN, via Crescentino snc, 13040 Saluggia, Vercelli, Italy). *B. burgdorferi* infection was not subsequently confirmed by a two-step process using recombinant and highly specific IgG-IgM line immunoassay (LIA) (*recom*Line IgG and IgM Borrelia tests, Mikrogen diagnostic; Ref. 4272 and 4273; Anna-Sigmund-Str. 10, 82061 Neuried, Germany).

Therefore, the diagnosis of borreliosis would have been possible only through direct microscopic detection of spirochetes in a peripheral blood smear, obtained at the height of fever and stained specifically with Wright’s or Giemsa stain [11]. We were unable to complete this diagnostic investigation phase because the acute phase had already passed, and the patient was already cured after the ABT cycle. Furthermore, the patient reported neither recent tick bites nor erythematous skin lesions indicative of an active infection. We therefore hypothesized the possibility that, the *B. recurrentis* infection vector might have been the *Pediculus corporis* [12], also considering the well-recognized occurrence of cross-reactivity among *Borrelia* spp. antigens.

Although CT images were not characteristic of worm colonization, such as *Strongyloides* infestation that could explain both the edema and hepatic abscesses [13], we performed a biomolecular assay for *S. stercoralis* that gave a negative result. Furthermore, we were able to exclude echinococcosis because plain radiographs and an abdominal ultrasound showed hepatomegaly, but not the characteristic scattered areas of hyperechoic lesions bordered by calcified rings, which were also not visible on CT. Furthermore the patient did not present eosinophilia or positivity to specific serum antibody test for *E. multilocularis* [14].

Therefore, considering the sequence of signs and symptoms revealed by the patient’s medical history—ranging from pediculosis to episodes of dysentery and Quincke’s edema—we also investigated other potential infections capable of causing diarrhea followed by the appearance of abscess-like hepatic lesions, sometimes accompanied by allergic manifestations. Starting from the hypothesis of close contact with people belonging to degraded environments, usually the only carriers of pediculosis corporis, we re-evaluated the anamnesis of the patient and he admitted that about a month before the onset of diarrhea, around mid-March, he had had paid sex with a non-Caucasian man.

Thus, we tested him for the most common sexually transmitted infections, such as HIV and *Treponema pallidum*, for which he tested negative. Given the rare forms of transmission involving liver abscesses, we hypothesized that this might be a case of invasive amoebiasis, also considering that the risk of intestinal *E. histolytica* infection is well documented in association with homosexual practices [15].

From an epidemiological perspective, two main and distinct species of *Entamoeba* are recognized: *E. histolytica*, which is highly pathogenic to humans, and *E. dispar*, a commensal species not associated with disease [16,17]. More recently, rare cases of cerebral abscesses were related to *E. moshkovskii* infection [18]. Considering that *E. histolytica* is transmitted via the fecal–oral route and can cause liver abscesses, amebiasis should be suspected in patients with an appropriate exposure history (residence in or travel to an endemic area) and characteristic clinical features, including fever, right upper quadrant pain, and marked hepatic tenderness [19,20].

Therefore, although amebiasis is not endemic in our region, we tested the patient’s blood for the research of specific antibodies against *E. histolytica*. Two independent serum samples had positive results, with an IgG-specific 1:1024 titer by ELISA (*Entamoeba histolytica* IgG ELISA; Ref. EIA-3830; DRG Instruments GmbH, Frauenbergstrasse, 18, 35039 Marburg, Germany).

**Figure 2:**
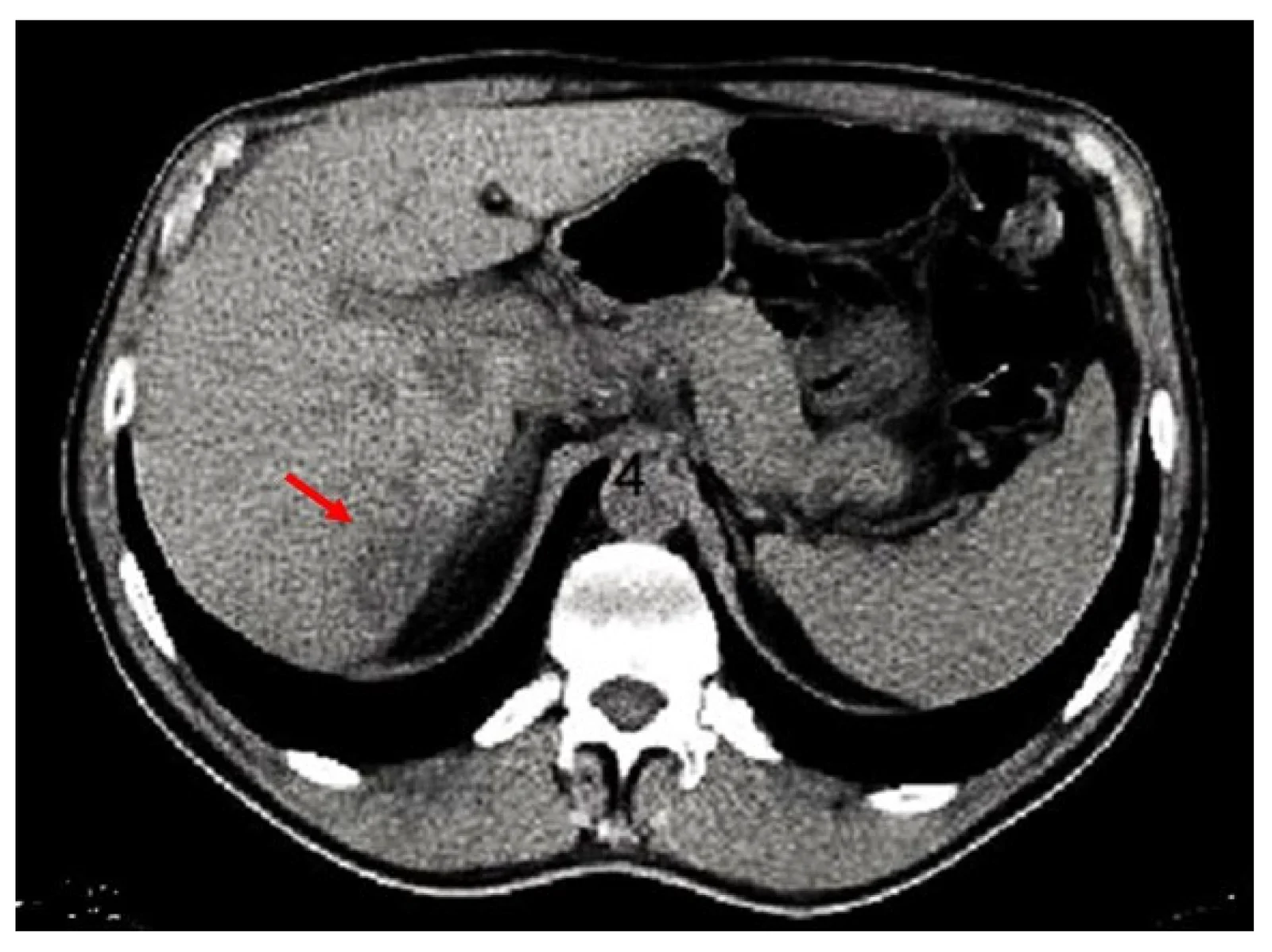
The larger liver lesion in segment 8 appears to have completely resolved, while the other lesion showed a significant reduction in size (red arrow) following effective antiparasitic treatment, as demonstrated by the CT performed 6 months later.

Thus, the patient was treated for *E. histolytica* with two cycles of metronidazole 750 mg tables and TID for 10 days, followed by paromomycin 30 mg/kg/day tablets in three doses daily, to be repeated after 20 days to eradicate luminal amoebae and prevent relapse. Patient evaluation after 6 months showed a decrease in *E. histolytica*-specific antibody titer (1:256) and CT images showed almost complete resolution of liver lesions (Figure 2).

Clinical case was resolved without complications, but we want to report some final considerations and limitations: (a) The patient received steroids to treat perioral edema and arthralgias, which are contraindicated in amoebiasis, because they can suppress the host’s immune response and likely facilitate the spread of the amoeba, especially during the acute intestinal phase. It is better to use acetaminophen 650 mg orally, to control symptoms or toxemia resulting from the use of spirochaeticidal; (b) The abscess puncture should always be avoided, because it is difficult to obtain a sample that is useful for diagnosis, as in our case, and furthermore, there are highly specific serological tests for diagnosis and very effective therapies for the treatment of the disease; and (c) Cross-reactions between antigens from *Borrelia* spp. can be observed, which can make *B. recurrentis* infection diagnosis more difficult if a specific microscopy identification or genetic test cannot be executed, as happened in this case.

## 3. Discussion

*Entamoeba histolytica* is the causative agent of amoebiasis—amoebic dysentery, one of the most common intestinal parasitic infections worldwide, accounting for an estimated 40 million infections and 100,000 deaths annually [1]. However, only 5–15% of infected individuals develop clinically manifested disease [2].

### 3.1. Diagnostic Protocols for Suspected Cases of Amoebiasis

The diagnosis of amoebiasis relies on parasite or cyst identification in stool or tissue by microscopy, or with the detection of specific serum antibodies by ELISA or of antigen by PCR [21]. During the acute phase, the presence of *E. histolytica* forms have the highest diagnostic sensitivity, although microscopy research in stool is highly dependent on the examiner’s expertise and identification in tissue is enhanced by periodic acid–Schiff (PAS) staining [22]. Despite its high specificity, PCR may be costly and not widely available [23], whereas ELISA antibodies may persist long after clinical recovery. In contrast, asymptomatic infections often remain undiagnosed, particularly in non-endemic countries where routine screening is not performed [1].

Therefore, in this complex case, a systematic clinical approach integrating multiple diagnostic clues was essential to confirm amoebiasis. These included identification of a relevant exposure history, temporal correlation between exposure and symptom onset, recognition of characteristic liver lesions, detection of specific serological markers, and confirmation through the patient’s favorable response to antiparasitic therapy.

### 3.2. Signs and Symptoms of Systemic Amoebiasis

The hepatic abscess, as found in this case, must be considered the first extraintestinal form related to amoebiasis [1]. It is predominant in young males by about 10:1 [24], although pregnant women and children are more prone to developing the infection [25]. Amoebic liver abscesses, which are highly endemic in most developing tropical countries, are now more frequently encountered in other geographic non-endemic areas [3]. Therefore clinical manifestations of amebiasis can range from asymptomatic carriers without disease to severe invasive forms, such as tissue abscesses (hepatic, cerebral, pulmonary) or fulminant colitis with paralytic ileus and intestinal perforation [26,27,28].

Occasionally, abscess aspiration is required to exclude a pyogenic liver abscess, whereas aspiration of an amoebic abscess is not routinely performed for diagnostic purposes because its core consists of liquefactive necrotic tissue. Unlike pyogenic liver abscesses, which are primarily managed with percutaneous or surgical drainage, amoebic liver abscesses are generally treated with first-line medical therapy [29]. Antiparasitic therapy, most commonly with metronidazole [30], is usually effective in treating amoebic dysentery and liver abscesses. Although clinical resolution is generally rapid, the complete radiological resolution of the abscess may take months to years.

*E. histolytica* is classified as a Category B biodefense agent because of its environmental stability, resistance to chlorination, and ability to spread through contaminated food or water in settings with poor sanitation, as well as through sexual transmission.

Abdominal ultrasonography, CT, and MRI may help identify a hypodense hepatic lesion, although an amebic liver abscess can sometimes be difficult to distinguish from other types of hepatic abscesses [31]. The differential diagnosis of a hepatic mass should include pyogenic abscesses and/or necrotic hepatocellular carcinoma (HCC), as well as amebic liver abscesses (Table 2), as reported in the literature from tropical regions [32,33,34].

### 3.3. Clinical Characteristics and Diagnostic Difficulties of Borrelia spp. Infections

**Figure 3:**
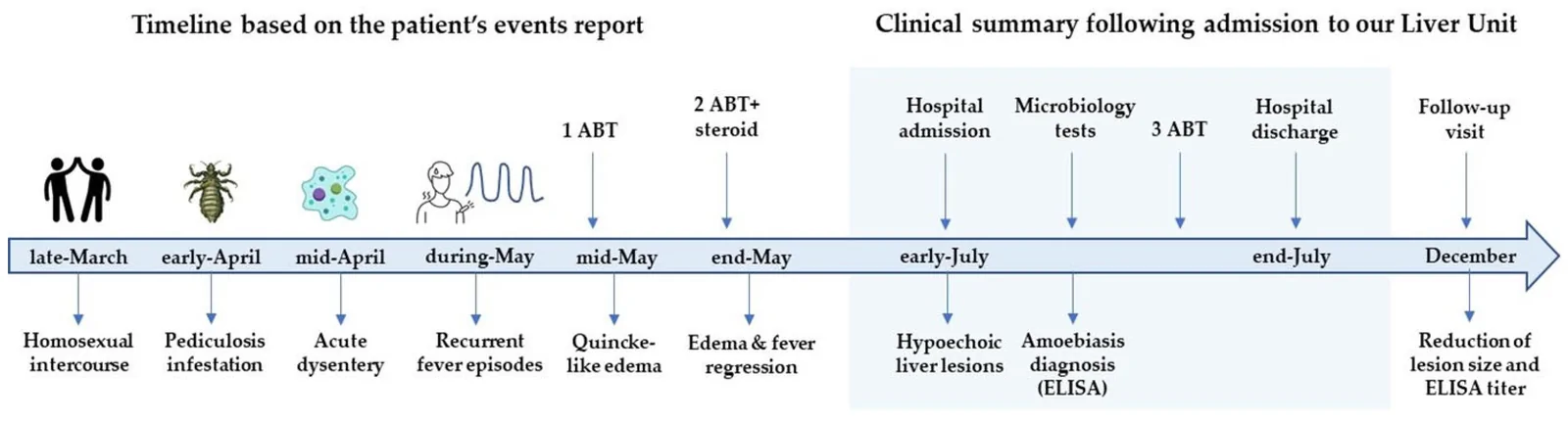
Chronology of clinical events as reported before and after hospital admission.

Returning to some considerations regarding the clinical case, we believe that the time-sequence of events were crucial to solving this diagnostic puzzle (Figure 3).

Given that pediculosis developed approximately two weeks after the presumed exposure and an episode of hemorrhagic dysentery occurred two weeks later, we investigated the possibility of a common risk factor. The temporal sequence and concomitant occurrence of ecto- and endo-parasitic infections raised the possibility of shared exposure, potentially suggesting sexual transmission. Nevertheless, this hypothesis cannot be confirmed, as the route of transmission remains uncertain, but highly probable.

Subsequently, the recurrent febrile state, characterized by episodes lasting 3–4 days alternating with remission phases of approximately one week—likely mimicked a form of RF. *B. recurrentis*, transmitted by lice, and *B. miyamotoi*, carried by ticks of the *Ixodes* genus—might have been the most likely causative agents of RF in this case. Unfortunately, it was not possible to diagnose the infection using serological methods. However, the etiological diagnosis of RF—based on a *Borrelia* spp.-specific CLIA test—might have been influenced by either false-positive results (due to other spirochetal infections, including LB) or considering that the patient had already recovered from the infection following prior specific antibiotic therapy. We are confident in the future availability of specific and sensitive diagnostic tools for RF causative agents by novel immunoassays, which are based on the N-terminal CihC and GlpQ fragment antigens that are highly specific to *B. recurrentis* infection [35], to overcome the difficulties of performing specific “in vivo” methods to detect *B. recurrentis* [8].

Furthermore, *B. burgdorferi* infection—the most widespread tick-borne disease endemic to northern Italy [36,37]—has not yet been isolated in body lice. The clinical manifestations of Lyme disease do not allow for an easy diagnosis of the infection, as it is a condition characterized by low-level bacteremia and more insidious, prolonged symptoms. Conversely, it is typically marked by a “bull’s-eye” rash or erythema migrans (EM) [38]—a sign never reported in this patient. However, occasional cases of viral-like febrile illness without EM have been attributed to LB, especially after spontaneous resolution of an unrecognized asymptomatic EM [39].

In conclusion, the patient could have experienced a typical episode of RF, probably related to *Borrelia* spp. infection, as suggested only by clinical features, temporal sequence of events, and the subsequent onset of a typical Jarisch–Herxheimer (JH) reaction [40]. This type of angioedema has been associated with the release of spirochete toxins and cellular cytokines, resulting in swelling of the tissues and mucous membranes, typically without the pruritus characteristic of Quincke-type angioedema. In this case, the reaction occurred shortly after the initiation of antibiotic therapy, consistent with the bactericidal activity, which typically begins within approximately 8–12 h after the dose intake.

## 4. Conclusions

In conclusion, we described a rare case of concurrent borreliosis and invasive amebiasis, presumably sexually acquired, presenting with hepatic abscesses. Such infections may reach non-endemic regions through population migration from areas where these pathogens are prevalent. Clinicians and general practitioners should be aware that fecal–oral pathogens may also be transmitted during sexual practices, including heterosexual and/or homosexual encounters, and may therefore present as sexually associated infections. Targeted screening of individuals who are at increased risk may help to identify and treat asymptomatic carriers and prevent further transmission.

The diagnosis and etiological characterization of *Borrelia* spp.-associated RF may be challenging. Diagnostic difficulties arise from serological cross-reactivity among *Borrelia* species, which may complicate the identification of the causative pathogen, as well as from the ability of *B. recurrentis* to undergo antigenic variation in its surface proteins, allowing immune evasion. This phenomenon contributes to the characteristic relapsing clinical pattern observed in infected individuals.

## 5. Key Points

✓ Effective antiparasitic therapy can rapidly eliminate *E. histolytica* trophozoites from infected tissues and eradicate cystic forms from the intestinal lumen.
✓ Acute *E. histolytica* infection typically presents with bloody diarrhea 2–3 weeks after infection, lasting 2–3 weeks, often accompanied by abdominal pain, flatulence, tenesmus, mild-to-moderate fever, and night sweats.
✓ Painful hepatomegaly, elevated ALT levels, and mild hyperbilirubinemia are typical findings of chronic, relapsing extraintestinal amebiasis.
✓ Diagnosis relies on imaging evidence of a hypodense focal liver lesion on ultrasound, CT, or MRI, supported by detection of *E. histolytica*-specific antigen or DNA in stool and/or positive serum antibodies.
✓ Body lice may transmit *B. recurrentis*, causing RF and, rarely, a Jarisch–Herxheimer reaction shortly after initiation of antibiotic therapy.
✓ *B. recurrentis* is diagnosed by identification of the spirochete in thick blood smears and, when necessary, confirmed by species-specific molecular methods.

## Figures and Tables

**Table 1:** Biochemical, hematological, microbiological and molecular test results.

Biochemical	Hematological	Microbiological
AST (U/L) 104 (<45)	WBC (10^9^/L) 15.5 (5–10)	Hepatitis virus (HBV, HCV) NEG.
ALT (U/L) 188 (<55)	Neutrophils (10^9^/L) 8.5 (<7)	HIV marker NEG.
GGT (U/L) 295 (<65)	Eosinophils (10^9^/L) 0.2 (<0.5)	*T. pallidum* ELISA NEG.
AP (U/L) 186 (<100)	HB (g/dL) 12.1 (14–16)	Fecal parasites NEG. (6 samples)
LDH (U/L) 247 (<120)	PLTS (10^9^/L) 480 (<450)	*Echinococcus* Ig Ab NEG.
T-Bilirub. (µmol/L) 25.2 (<17)	CRP (mg/L) 15 (<5)	Blood/Urine cultures NEG.
D-Bilirub. (µmol/L) 4.9 (<3)	C1-inhibitor level NORMAL	*Chlamydia* PCR/NAAT NEG.
Creatinine (µmol/L) 66 (<90)	C2 and C4 proteins NORMAL	*S. stercoralis* ELISA NEG.

**Table 2:** Differential diagnosis of various types of liver lesions which tend to form abscesses.

	Pyogenic	Amoebic	HCC
Favorable Risk Condition	Recent endovascular proce- dures or surgical operations. Subject with immunodeficiency or immunosuppressed.	Staying in endemic areas. Close contact with immigrants or travelers from regions at risk of parasitic infestation.	Presence of chronic liver disease and/or liver cirrhosis. Positive hepatitis viral markers. Drug and/or alcohol abuse.
Imaging Features (T2-weighted, delayed portal-phase images)	Single or multiple, roundish, hypointense lesion, sign of edema and inflammation, with a hyperintense peripheral margin or capsule and a hypodense center, as in a necrotic lesion.	Single or multiple irregular hypointense lesion with a rim of connective tissue forming a soft capsule with parasites nestled within, which delineates inflammatory and sometimes necrotic tissue in the core.	Single or multiple rounded hypointense lesion with specific liver contrast wash-out, and hyperintense ring (or APHE capsule), DWI positive. Sometimes with necrotic central evolution.
Histological Pattern	Acute–subacute lesion with central liquefactive necrosis (pyocytes-rich material) in a context of liver tissue only partly degraded and invaded by inflammatory (WBC, lymphocytes and phagocytic) cells. Surrounded by a connective tissue capsule.	Subacute–chronic lesion with diffuse liquefactive liver tissue rich in macrophages or histiocytes, filled with RBC, WBC, lymphocytes and cell debris. Resembling anchovy paste-like contents, included by soft connective tissue.	Well-differentiated malignant hepatocytes (HSP70/GS/GPC3 positive at IHC), interdigitate with hepatocytes arranged in a lobular architecture interrupting by thin fibrosis septa and with extensive infiltration of lymphocytes, WBC and macrophages.

Abbreviations: HCC, hepatocellular carcinoma; WBC, white blood cells; RBC, red blood cell; DWI, diffusion-weighted imaging; APHE, arterial-phase hyperenhancement; IHC, immunohistochemistry staining; HSP70, heat shock protein 70; GPC3, glypican protein 3 and GS, glutamine synthetase.

## Data Availability

The data presented in this study are available from the corresponding author upon reasonable request, in accordance with the General Data Protection Regulation (GDPR).

## Abbreviations

The following abbreviations are used in this manuscript:

ABT	Antibiotic therapy
AP	Alkaline phosphatase
ALT	Alanine amino-transferase
APHE	Arterial-phase hyperenhancement
AST	Aspartate amino-transferase
C1, C2, C4	Complement C1, C2, C4 components
CLIA	Chemiluminescence assay
CPR	C protein reactive
CT	Computer tomography scan
DWI	Diffusion-weighted images
ELISA	Enzyme-linked immunosorbent assay
EM	Erythema migrans
GGT	Gamma-glutamil transferase
GP	General practitioner
HB	Hemoglobin
HBV	Human hepatitis B virus
HCC	Hepatocellular carcinoma
HCV	Human hepatitis c virus
HIV	Human immunodeficiency virus
IHC	Immunohistochemical
JH	Jarisch Herxheimer reaction
LB	Lyme borreliosis
LDH	Lattic dehydrogenase
LIA	Line-recombinant immunoassay
MR	Magnetic resonance
NAAT	Nucleic acid amplification test
PCR	Polymerase chain reaction
PLTS	Platelets count
RBC	Red blood count
RF	Relapsing fever
RT	Reverse transcriptasi
spp.	Species
STE	Sexually transmitted enteric disease
TID	Two intakes daily
US	Ultrasound examination
WBC	White blood count
